# Feeding a Bitter Mix of Gentian and Grape Seed Extracts with Caffeine Reduces Appetite and Body Fat Deposition and Improves Meat Colour in Pigs

**DOI:** 10.3390/ani15142129

**Published:** 2025-07-18

**Authors:** Maximiliano Müller, Xinle Tan, Fan Liu, Marta Navarro, Louwrens C. Hoffman, Eugeni Roura

**Affiliations:** 1 Queensland Alliance for Agriculture and Food Innovation, The University of Queensland, Brisbane, QLD 4072, Australia; m.mullerbravo@uq.edu.au (M.M.); xinle.tan@uq.edu.au (X.T.); m.navarrogomez@uq.edu.au (M.N.); louwrens.hoffman@uq.edu.au (L.C.H.); 2 JBS Pork Australia Pty Ltd., Corowa, NSW 2646, Australia; fliu@rivalea.com.au

**Keywords:** backfat, bitter compounds, feed intake, plant extracts, pork quality

## Abstract

Dietary bitter compounds such as caffeine have the potential to reduce backfat in pigs. However, the use of caffeine as a feed additive has restrictions in many countries. It was hypothesised that grape seed and gentian plant extracts (GG) could replace caffeine in feed due to their bitterness and antiadipogenic effects. The effect of caffeine (0.5 g/kg), GG (2 g/kg) alone or in combination with caffeine (BM) at increasing concentrations (0.5, 1, 1.5, or 2 g/kg) on feed efficiency, carcass, and meat quality was assessed in finishing pigs (Large White × Landrace). Growth performance and carcass traits were evaluated at a pen level (n = 14). Loins (longissimus thoracis) were removed from eight pig/treatment at the abattoir to assess drip loss, lightness (L*), redness (a*), yellowness (b*), chroma (C*), hue angle (h°), pH, cook loss, and shear force. A linear increase (*p* < 0.05) in loin a*, b*, and C* values and a linear decrease (*p* < 0.05) in ADFI, ADG, backfat, dressing percentage, and HSCW were observed with increasing BM levels. At 1.5 g/kg, BM increased the loins a* (*p* < 0.05), b* (*p* < 0.05) and C* values (*p* < 0.05) compared to the control. Twenty-two proteins related to energy metabolism and myofibril assembly were identified to be upregulated (FDR < 0.05) in BM vs. control loins. In conclusion, GG could be used in combination with low doses of caffeine to modulate appetite and carcass leanness and improve pork colour.

## Simple Summary

Excessive fat content in pork negatively impacts consumer choice. Bitter compounds, such as caffeine, can be used to prevent excessive fat deposition in pigs. However, the use of caffeine as a feed additive has restrictions in several countries. Plant bitter extracts, such as grape seed and gentian, have the potential to be used as a replacement for caffeine due to their capacity to reduce fat tissue development and appetite. The aim of this study was to assess the effects of a gentian and grape seed extract mixture, alone or in combination with caffeine (BM), at increasing doses on the growth performance, carcass traits, and meat quality of pigs. The results showed that the plant bitter extracts alone were not effective in reducing fat deposition. However, when combined with low doses of caffeine, these bitter compounds reduced the feed intake, increased carcass leanness, and improved the pork colour by intensifying the meat redness and yellowness. Increasing the dietary levels of BM linearly reduced carcass traits (i.e., backfat and weight) and growth performance (feed intake and weight gain), and increased meat colour intensity. The meat colour changes of BM-fed pigs were associated with changes in the abundance of energy metabolism and muscle structure proteins

## 1. Introduction

One of the main drivers in pork consumer choice relates to flavour and appearance (e.g., colour and fat cover) which are features associated with product quality [1,2]. Finishing pigs are voracious animals that generally consume feed above their requirement for optimal growth, resulting in poor efficiency and excess carcass fat associated with an undesirable appearance for consumers [3,4]. Pork fattiness and paleness are known deterrents for consumers [4] In addition, high carcass fat is synonymous with low feed efficiency and elevated production costs [5]. Metabolic modifiers such as somatotropin or ractopamine have been shown to produce leaner carcasses and improved growth efficiency [6,7,8]. However, their use is restricted in multiple countries due to regulatory policies and a consumer preference for hormone and drug-free meat production [9,10]. Thus, alternative approaches are needed to reduce carcass fat. This is particularly important in markets such as Australia, Europe, and the United States, where producers are penalised when supplying carcasses with high backfat [11,12,13].

Caffeine has been shown to be an effective alternative to ractopamine as it stimulates fat mobilization and carcass leanness in pigs [14]. However, undesirable dose-dependent effects resulting in skin rash, retarded growth, and increased mortality, have been reported [15,16]. In addition, policies in Western countries limit caffeine usage in food, indicating that potential limitations around the use of caffeine as a feed additive (e.g., maximum allowed doses) may also arise in the future, highlighting the need for safe and cost-effective alternatives [17,18].

The effect of caffeine on carcass leanness has been associated in part with its capacity to restrict appetite [16]. Like caffeine, several plant compounds have been shown to reduce feed intake in pigs associated with their high levels of bitter compounds such as glucosinolates and phenols [19,20,21]. This, in turn, would lead to a decreased energy intake, affecting fat deposition. Previous research evaluated the preference for 16 natural, non-toxic compounds known to be bitter to humans, showing that gentian and grape seed extracts (GG) were rejected compared to plain water in a double-choice paradigm showing potential to modulate feed intake in pigs [22]. In addition, GG have strong antioxidant properties due to their high content of phenolic compounds (e.g., proanthocyanidins and xanthones) which, can enhance the oxidative stability of meat and, therefore, its sensory attributes (e.g., flavour and colour) by scavenging and neutralizing free radicals derived from lipid oxidation [23,24,25]. Thus, GG have the potential to improve feed efficiency, backfat deposition, and pork quality in finishing pigs.

This study aimed to assess the effect of the dietary inclusion of bitter plant extracts, gentian and grape seed (main chemical compounds: gentiopicroside and proanthocyanidin, respectively), in a partial or total substitution of caffeine, on feed efficiency as well as carcass and meat quality in finishing pigs. It was hypothesized that the plant extracts would reduce feed intake and P2 backfat to a similar extent as caffeine while improving feed conversion and meat colour.

## 2. Material and Methods

### 2.1. Animals, Housing, and Diets

The experiment was performed at the Rivalea’s Research & Innovation grower/finisher shed in Corowa, New South Wales, Australia. A total of 98 pens (14 pigs/pen) with Large White × Landrace pigs (initial weight 66.55 ± 7.47 kg) were used. Pigs were distributed into pens based on weight and sex, across 3 experimental runs (consisting of 28, 42, and 28 pens in runs 1, 2, and 3, respectively, with a 24 h elapsed time between the entry of each run) and given 1 of 7 dietary treatments equally replicated in each run (4 pens/treatments for run 1 and 3, and 6 pens/treatment for run 2): a commercial standard finisher diet (control), the standard diet supplemented with 0.5 g/kg of caffeine, the standard diet supplemented with GG (Xi’an Pincredit Bio-tech Co., Ltd, Shaanxi, China) at 1.5 g/kg, or a bitter mix (BM) (combination of the bitter plant extracts (75%) and caffeine (25%)) at 0.5, 1, 1.5, or 2 g/kg (Table 1). The treatments were randomly distributed within sex and weight categories (light or heavy). Pigs had ad libitum access to feed and water for the duration (42 d) of the experiment.

### 2.2. Growth Performance and Carcass Traits

Performance parameters including, average daily feed intake (ADFI), average daily gain (ADG), and feed conversion ratio (FCR), were calculated on days 21 and 42 of the experiment. Feed intake was estimated by measuring feed disappearance, whereas the ADG of the pigs was calculated based on the pen’s weight at days 0, 21, and 42. At the end of the experiment, the animals were transported to a commercial abattoir located within 1 km distance from the research farm. Pigs were gas stunned with CO_2_ before slaughter following a 24 h lairage. The hot standard carcass weight (HSCW) was measured immediately after dressing (Trim 1, AUS-MEAT Ltd., Corowa, Australia). Loin muscle and backfat depth were measured at the P2 site (65 mm from the midline over the last rib) in all pigs within each pen using the Hennessy Chong fat depth probe (used as well to calculate carcass lean yield [26]. The average dressing percentage was calculated using the pen average body weight at sale and carcass weight measurements.

### 2.3. Meat Quality Assessment

Meat quality was assessed in 8 pens per treatment (1 pig/pen), which were randomly selected across runs. The selection of animals within pens for meat quality measurements was based on a visual assessment of their body condition to ensure that they were representative of the pen. The number of animals selected for meat quality followed the criteria used in a previous study [27]. The meat quality assessment included the evaluation of the longissimus thoracis muscles’ (from here on referred to as loins) drip loss %, colour (lightness (L*), redness (a*), and yellowness (b*)), pH (45 min and 24 h post slaughter), cook loss %, and Warner–Bratzler shear force. The loin cut was separated from the shoulder at the 5th rib from the left side of each pig carcass in the boning line. Each loin was orientated caudal to cranial and trimmed by removing and discarding 100 mm from the caudal end. The remaining 25-, 40-, and 60-mm loin samples from the caudal end were used for colour, drip loss, and shear force measurements, respectively. The loin samples underwent processing as a single batch for the assessment of meat quality.

The pH of the loin was determined adjacent to the P2 site at 45 min and 24 h post slaughter (samples stored at 2 °C before measurements) using a portable meat pH metre (Model HI98163, Hanna Instruments, Woonsocket, RI, USA) fitted with a polypropylene spear-type gel electrode and a temperature probe (Model FC2323, Hanna Instruments, Woonsocket, RI, USA). The pH meter was calibrated using three buffer solutions with a pH of 4.01, 7.01, and 10.01 (Buffer Solution, Hanna Instruments, Woonsocket, RI, USA) at 25 °C.

Pork colour (L*, a*, and b*) was measured in triplicate on each loin steak’s anterior face after 10 min of blooming at 20 °C using a Minolta Colour Chromameter CR-400 (Minolta, Osaka, Japan) set at an aperture of 8 mm calibrated on a white tile with D65 illumination and a 2° standard observer. Chroma (C*) and hue angle (h°) values were calculated using the equations = √a*^2^ + b*^2^ and arctangent (b*/a*), respectively. Colour measurements in the loin samples were performed following a 24 h storage period at 2 °C.

Drip loss on loin samples chilled overnight at 2 °C was determined using the suspension method [28]. In brief, the loin muscle was trimmed of all fat and sinew before being cut into cubes (40 × 40 × 40 mm) of average weight of 65 g (initial weight). The cube samples were weighed and placed in a nylon net (20 × 20 mm squares) in a polypropylene container and secured by a lid screwed over the netting. Samples were then transferred into a refrigerator set at 2 °C and stored for 24 h before having their excess moisture removed with absorbent paper and their weight recorded. Drip loss was subsequently calculated, and the weight loss was expressed as a percentage of the initial weight.

For the evaluation of cooking loss and meat shear force, loin samples were processed following a slightly modified methodology described by Bouton et al. [29] and Bouton and Harris [30], respectively. In short, frozen loin samples (60 × 50 × 40 mm) of average weight of 75 g, previously stored in zip-lock bags at −20 °C for 14 d, were hung on a rack before being immersed in a water bath at 70 °C for 30 min and then immediately placed in ice cold water for 35 min. The cooled loin samples were removed from their sealed bag, patted dry with absorbent paper and re-weighed to determine their cooking loss percentage. The cooked loin samples were then placed on a cold tray (previously refrigerated at 2 °C for 24 h) wrapped in plastic film for 10 min before being cut into 10 mm thick strips (>30 mm long) parallel to the muscle fibres. Meat samples (6 replicates) were placed in a Warner–Bratzler attachment (isosceles triangle with a side length of 40 mm and blade thickness of 1 mm) fitted to a shear force measurement equipment (Mecmesin^®^ BFG 500N, Slinfold, UK) to evaluate the average force (N) required to shear the samples perpendicular to the fibres’ length. The vertical cut of the meat samples was performed in an area of 10 × 10 mm at a speed of 200 mm/min.

### 2.4. Proteomics

#### 2.4.1. Sample Preparation

Pork loin samples (n = 8/treatment) were prepared following the filter-aided sample preparation method as described by Wiśniewski [31]. Briefly, 100 mg of tissue from the control and BM 1.5 g/kg loin samples (stored at −20 °C) were cut and placed in an Eppendorf tube. The tissues were immediately denatured by Guanidine (6 M, 50 mM Tris-HCl, pH = 8.5), reduced by Dithiothreitol (40 mM), alkylated (20 mM Acrylamide), and cleaned by Molecular Weight Cut Off tubes (10 kDa) to remove detergents by centrifugation at 14,000× *g* for 40 min. The cleaned tissues were subsequently digested with Trypsin (Trypsin Gold, Mass Spectrometry Grade) (Promega, WI, USA) in Ammonium Acetate buffer (100 mM, pH = 7.0) overnight and desalted by C18 ZipTip (Millipore, NSW, Australia) before Mass Spectrometry analysis. The obtained peptides were analysed by Zeno time-of-flight (ZenoTOF) (Sciex, Framingham, MA, USA) in Sequential Windowed Acquisition of all Theoretical fragment ions (SWATH) mode followed by data-independent acquisition neural network (DIA-NN, version 1.9.2) for library-free identification and quantified by MSstats for differential protein characterization [32,33,34] as detailed below.

#### 2.4.2. Mass Spectrometry

The C18 ZipTips desalted peptides were separated using reversed-phase chromatography on a Waters M-Class Ultra-performance liquid chromatography system. The samples were loaded onto a Waters NanoEase HSS T3 column (100 A, 1.8 um, 300 um × 150 mm). Chromatography was performed at 5 uL/min and column set at 40 °C, with liquid chromatography conditions as follows: 0–0.6 min = 5% acetonitrile with 0.1% formic acid, 0.6–22 min = 5–35%, 22–23 min = 35–90%, held at 90% for 3 min followed by re-equilibration for 4 min. Eluted peptides were directly analysed on a ZenoTOF 7600 instrument (Sciex, MA, USA) using an OptiFlow Micro/MicroCal source. Curtain gas = 35 psi, CAD gas = 7 psi, Gas 1 = 20 psi, Gas 2 = 15 psi, source temp = 150 °C, spray voltage = 5000 V, DP = 80, CE = 10.

For Zeno-SWATH acquisitions, an MS TOF scan across 400–1500 *m*/*z* was performed (0.1 s). For MS2, variable windows spanning 399.5–750.5 *m*/*z* were chosen for fragmentation (0.013 s) with fragment data acquired across 140–1750 *m*/*z* with Zeno pulsing on, and a threshold set to 100,000 cps. Collision-induced dissociation was used for fragmentation.

#### 2.4.3. Protein Identification

Peptides and proteins were identified using DIA-NN, by searching against the porcine proteome (downloaded from UniProt, Proteome ID: UP000008227) in library free mode. The settings allowed up to two tryptic missed cleavages and a maximum of three variable modifications/peptide. A spectral library was created from the DIA data and was subjected to PeakView 2.1 (Sciex, Framingham, MA, USA) quantification, with the following setting: shared peptides (allowed), peptide confidence threshold (99%), false discovery rate (1%), XIC extraction window (6 min), XIC width (75 ppm). The obtained output was fed to the ms2go (https://github.com/bschulzlab/ms2go (accessed on 2 December 2023)) program for protein difference analysis and Gene Ontology (GO) term enrichment.

### 2.5. Statistical Analysis

The effects of increased dietary concentrations of BM on performance and carcass parameters were analysed by regression analysis using a linear and quadratic model in R4.3.2 software (RStudio, Inc., Boston, MA, USA). The dose response effect of the BM on performance and meat quality parameters considered “Run”, “Sex”, and “Initial weight” (the last one only for performance traits) as factors in the model. Treatment comparisons for performance and carcass parameters used the pen as an experimental unit, including the group weight and feed intake measures. To determine the individual and combined effects of the plant extracts and caffeine (comparison included control, caffeine, BM 2.0 g/kg and GG treatments) on performance and carcass traits, an Analysis of Variance (ANOVA) considering “Run”, “Sex”, “Treatment”, “Initial weight” and the interaction between “Sex” and “Treatment” as factors, followed by a Tukey multiple comparison, test was used. For meat quality measures, pens were randomly selected across the 3 runs. The ANOVA used to determine the individual and combined effects of the plant extracts and caffeine (4 treatments previously described) on pork quality measures included the factors “Sex” and “Treatment” and the interaction between these two factors, which was then followed by a Tukey multiple comparison test. The “Run” effect was not included in the ANOVA model for pork quality measures as the specific pen number of the selected carcasses were not recorded. Therefore, pen details, such as light or heavy weights, were not included in this analysis. To compare the effect of all bitter compound treatments to the control diet, the same ANOVA model previously described was used for meat quality measures, followed by a Dunnett multiple comparison test. A correlation analysis between loin colours and pH 24 h postmortem was performed using Pearson’s correlation and the data residuals to account for treatment variance. In all models, only a single measurement from each pen was used. This was either a mean when multiple animals were measured or data for a single pig (i.e., meat quality data). Performance, meat quality, and carcass parameters were considered statistically significant at *p <* 0.05. Adjusted *p*-values for the proteomics and GO term enrichment data were -Log10 transformed. The protein difference analysis and GO term enrichment adjusted *p*-value cut off was 0.00001 and 0.01, respectively.

## 3. Results

The effect of the dietary bitter compounds (GG, caffeine or their mixture (BM)) on the growth performance in finishing pigs is shown in Table 2. The BM (2 g/kg) significantly decreased (*p* < 0.01) ADFI during the first 3 weeks of the experiment when compared to the control or the GG treatment. In the overall period (day 0 to 42), a significant (*p* < 0.01) reduction in ADG was observed in the caffeine and the 2 g/kg BM when compared to the control and GG groups. When evaluating the dose response effect of the BM on pig performance (Table 3), a linear decrease (*p* < 0.05) in ADFI and ADG was identified over the total period with increased doses of the BM in the diet. Similarly, a linear reduction in ADFI (*p* < 0.001) and ADG (*p* < 0.05) and a quadratic effect on ADG (*p* < 0.01) were observed during weeks 1 to 3. During weeks 4 to 6, only a linear decrease in ADG (*p* < 0.05) was identified with increased doses of the BM.

Pigs fed caffeine or the BM at 2 g/kg diet had lower P2 backfat (*p* < 0.05) compared to the control group (Table 2). Similarly, a significant linear decrease (*p* < 0.05) in the HSCW, P2 backfat and dressing percentage was identified with increasing concentrations of the BM (Table 3).

**Figure 1:**
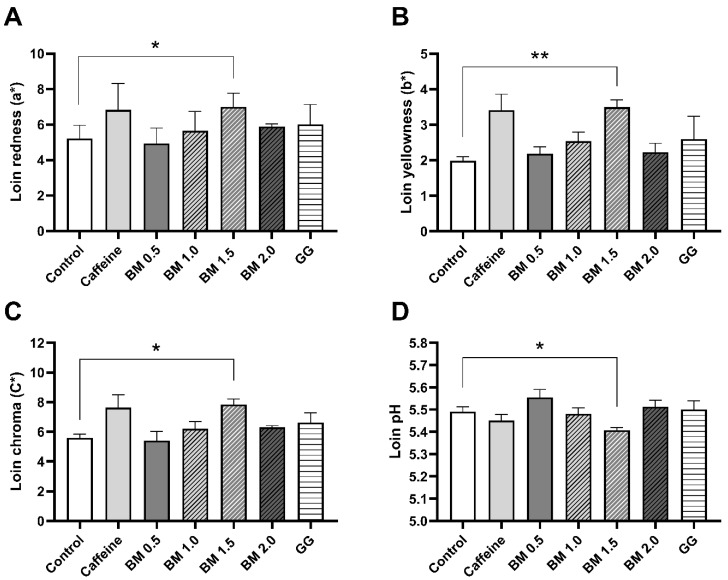
Effect of bitter compounds on pork quality attributes. Loin (longissimus thoracis *muscle*) redness (a*) (**A**), yellowness (b*) (**B**), chroma (C*) (**C**) and ultimate pH (**D**) values in pigs fed a control, caffeine (0.5 g/kg), gentian and grape seed extract (GG; 1.5 g/kg) or a bitter mix (BM; caffeine + gentian plant + grape seed extract) at 0.5, 1, 1.5, and 2 g/kg during the finisher phase. n = 8, each data point within the boxplot represents an individual pig. * = *p* < 0.05; ** = *p* < 0.01.

**Figure 2:**
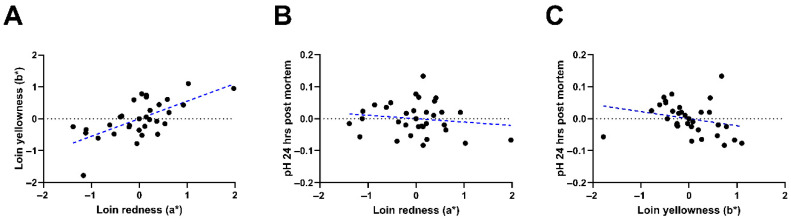
Association of pork quality attributes in pigs fed bitter compounds. Correlation analyses of loin (longissimus thoracis *muscle*) redness (a*) and yellowness (b*) (**A**), loin a* and b* vs. pH 24 h after slaughter ((**B**) and (**C**), respectively) of finishing pigs fed dietary bitter compounds (caffeine, gentian plant + grape seed extract or a mixture of the three) using data residuals. n = 8. (**A**): R^2^ = 0.4282, *p* < 0.001, (**B**): R^2^ = −0.0212, *p* > 0.05, (**C**): R^2^ = −0.0647, *p* > 0.05.

Pork quality results are presented in Table 4 and Table 5. The a* (*p* < 0.05), b* (*p* < 0.05), and C* values (*p* < 0.05) of the samples increased linearly with BM levels, whereas a quadratic dose response (*p* < 0.05) was observed for h° measurements. The BM at 1.5 g/kg increased the a* (*p* < 0.05), b* (*p* < 0.01), C* (*p* < 0.05), while also lowering the pH 24 h postmortem (*p* < 0.05) of the loin when compared to the control treatment (Figure 1). In addition, a moderate positive correlation was observed between the a* and b* (r = 0.655, *p* < 0.001) (Figure 2A), but not between the pH measured 24 h after slaughter and both the a* (r = −0.145, *p* > 0.05), and the b* of the loin (r = −0.254, *p* > 0.05) (Figure 2B,C). Results on drip loss percentage, cook loss percentage, shear force, L* value, pH 45 min and 24 h postmortem showed no significant treatment and BM dose-response effects (*p* > 0.05), respectively.

**Figure 3:**
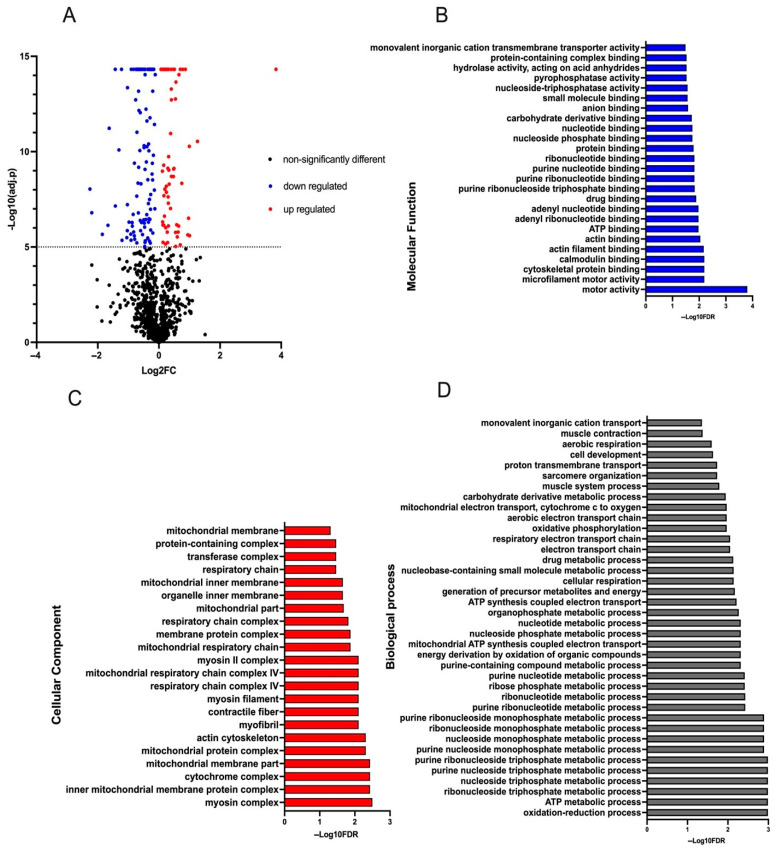
Effect of bitter compounds on the pork proteome. Characteristics of the identified differentially abundant proteins (DAP) between the loin (longissimus thoracis muscle) of finishing pigs fed a bitter mix (caffeine + gentian plant + grape seed extract) at 1.5 g/kg and a standard commercial diet (control). (**A**): Volcano diagram of quantified proteins; (**B**–**D**): Gene Ontology terms enriched from the DAP. −Log10 (adjusted *p*-value) was plotted against Log2 (Fold Change) of the identified proteins. Adjusted *p*-value of 0 was set to the next lowest value for plotting purpose in this graph. For the Gene Ontology terms, significance (based on −Log10 transformed values) was determined at an FDR < 0.05.

Following the proteomics analysis, 1189 proteins were identified and quantified in the loin samples by SWATH combined with DIA-NN. Among them, 181 differentially abundant proteins (DAP) were reported (adjusted *p* < 0.00001), of which 64 were upregulated and 117 downregulated when comparing the BM at 1.5 g/kg treatment to the control group (Figure 3A). Using GOstats, 86 terms were found to be enriched, associated to mainly 22 upregulated DAP (FDR < 0.05) (Figure 3B–D and Supplementary Table S1). These GO terms were primarily related to energy metabolism, muscle system processes and contraction. Of these DAP, 13 were related to adenosine triphosphate (ATP) metabolic processes, from which two were associated with glycolysis (phosphorylase kinase (PHKG1) and ATP-dependent 6-phosphofructokinase (PFKM)), one to fatty acid beta-oxidation (short-chain specific acyl-CoA dehydrogenase, mitochondrial (ACADS)), nine to the mitochondrial aerobic respiratory chain (NADH dehydrogenase [ubiquinone], and one to each of the following: beta subcomplex subunit 8, mitochondrial (NDUFB8), cytochrome c oxidase subunit 5A, mitochondrial (LOC100156967), cytochrome b-c1 complex subunit 8 (UQCRQ), cytochrome c oxidase subunit 4 isoform 1, mitochondrial (COX4I1), cytochrome c oxidase subunit 5B, mitochondrial (COX5B), malate dehydrogenase, mitochondrial (MDH2), ATP synthase protein 8 (ATP8), ATP synthase subunit alpha, mitochondrial (ATP5F1A) and creatine kinase (CKMT2), and one to transmembrane transport (sodium/potassium-transporting ATPase subunit alpha-2 (ATP1A2)). In addition, six DAP were associated with muscle contraction/myofibril assembly (Myosin-2 (MYH2), Myosin-4 (MYH4), Myosin-6 (MYH6), Myosin-7 (MYH7), Myozenin-1 (MYOZ1), and Troponin T, slow skeletal muscle (TNNT1)), one to cytokinesis and intracellular protein transport (kinesin family member 20B (KIF20B), one to intracellular vesicle-mediated transport (Exocyst complex component Sec8 (EXOC4)) and one to cell proliferation and cell cycle progression (Myosin-16 (ENSSSCG00000035534, using the Ensemble data base).

## 4. Discussion

The inclusion of GG in combination with caffeine significantly reduced feed intake in the finishing pigs. The reduced appetite (linear response) was more robust at the higher doses of the BM tested. These results align with previous studies showing that plant bitter compounds, such as glucosinolates and polyphenols, decrease preferences and feed intake in pigs [21]. Gentian and grape seed extracts are known to be strongly bitter to pigs [22]. This, in turn, had an early impact, decreasing the ADFI during the first three weeks of the experiment. Pigs have been reported to have a high tolerance to non-toxic bitterants, which is consistent with the results observed in the second half of this study when no differences in ADFI were observed, denoting an adaptation process [35,36]. However, a sustained reduction in ADG was observed in pigs fed the BM (reduction being more pronounced at higher doses) and caffeine but not the GG treatment. Reduced growth rates linked to caffeine have been reported in finishing and neonatal pigs, suggesting that the lower ADG observed is related to increased metabolic rates associated with caffeine [16,37,38]. These observations are consistent with an increased metabolic rate in muscle cells indicated by the upregulation of ATP metabolism and mitochondrial related proteins in the loin of BM-fed pigs compared to the control group.

Backfat thickness is a carcass trait that has relevance in pig production because of the consumer demand for leaner pork cuts and the market penalizations for the supply of overly fat carcasses [11,13]. Backfat thickness at the P2 level was reduced in BM and caffeine-fed pigs when compared to the control group in the present study. Moreover, backfat thickness, dressing percentage and HSCW linearly decreased with higher doses of the BM. These results are consistent with previous work demonstrating that bitter compounds lower fat deposition in pigs [39,40,41,42]. Nonetheless, the non-significant reduction of P2 backfat noted in GG fed pigs indicates that caffeine was the main component in the BM limiting backfat deposition. Considering the similar ADFI levels observed across treatments during the latter half of the study, it could be speculated that the reduced backfat content in the BM-fed pigs might have been primarily associated with the fat mobilization capacity of caffeine, and to a lesser extent, the antiadipogenic effect of GG rather than a potential appetite modulatory effect caused by their bitterness [15,43,44]. Even though no GO terms were successfully enriched from the downregulated proteins in our study, several of these proteins were identified as being involved in cholesterol and fatty acid transport (e.g., fatty acid binding protein 5, apolipoprotein A-I, adipocyte fatty acid-binding protein), indicating a potential suppression of lipid deposition in the loin of BM pigs.

Meat colour is one of the key quality attributes that influence consumers’ purchase decisions as it is generally associated with the freshness and wholesomeness of the product [45]. In particular, consumers show avoidance for pork cuts with lighter/less intense colours [46]. Pork paleness and lightness are highly correlated with its initial and ultimate pH, as low pH values tend to lead to the shrinkage of muscle fibres and the loss of water holding capacity, leading to an increased amount of light scattered at the surface of the cut [47]. In our study, the loin pH (45 min and 24 h postmortem) was found to be within the normal range, indicating that the pigs were exposed to minimal ante mortem stress and that the carcasses did not suffer from the pale, soft and exudative condition [48]. However, an increase in a* and b* ordinates and the vividness of these colours (C* values) coupled with a reduction in ultimate pH was detected in BM (i.e., 1.5 g/kg of BM) vs. control loins. These differences in pork quality may be associated to the antioxidant properties of the plant extracts, their natural pigments, and/or their capacity to induce metabolic changes in muscle cells [49,50,51].

Our proteomics data indicates that BM loins had a higher abundance of oxidative muscle fibres (e.g., fibre types I and IIA) relative to the control group, which may explain their elevated content of mitochondrial aerobic respiratory chain-related proteins. The increased mitochondrial proteins are indicative of aerobic ATP synthesis in BM muscle cells postmortem. This metabolism is normally associated with lower lactic acid production and myoglobin oxidation, which together with the antioxidant effect of GG, could have led to the retention of the pork a* and the increased colour intensity observed in BM vs. control samples [49,52,53]. In addition, the increased pork b* observed in BM loins may be linked to changes in fatty acid composition (ratio of polyunsaturated to saturated fatty acids) facilitating their oxidation during storage and/or to a reduction in muscle fat deposition triggered by the combination of bitter compounds and caffeine [16,50,54]. The shift in colour towards yellow more than red may also explain the relative higher h° values identified in the BM at 1.5 g/kg samples when compared to the other doses tested. Interestingly, no significant differences in L* values between BM and control loin samples were found, despite the BM group exhibiting a lower ultimate pH and a higher abundance of glycolysis- related proteins and creatinine kinase, which are generally associated with muscle protein denaturation and a reduced water-binding capacity [55,56]. The relative higher abundance of oxidative muscle fibres and increased mitochondrial activity (indicative of higher oxygen and NADH reserves) in the BM loins, could have prevented a drop in the pH 24 h below 5.4, which is typically associated with increased pork paleness, even with the increased glycolytic activity identified in these samples [57,58,59]. Compared to L*, much less is known regarding the relationship between glycolysis, muscle enzyme activity, and a* and b* in pork. Therefore, the relationship between muscle ATP metabolism and pork a* and b* merits further study. Overall, our data indicate that the BM loin more intense colour is primarily associated with muscle changes in muscle structure and energy metabolism.

Myoglobin (Uniprot accession: P02189) has been identified as the main red pigment in meat. It is generally abundant within oxidative muscle fibres, but it was not significantly different in the BM loin samples compared to the control [60]. Most proteomic studies to date show a similar dissociation between myoglobin abundance and pork colour [61,62,63]. The myoglobin content in pork has been shown to be lower than that of more intensely red-coloured meats, such as beef, lamb, or deer [64]. In addition, pork myoglobin seems to be less susceptible to lipid oxidation when compared to other red meats, suggesting structural differences [65]. The relevance of myoglobin in pork colour stability and colour saturation should be further explored. Future studies should focus on the direct measurement of myoglobin and its relationship with pork redness.

In the present study, the levels of gentiopicroside, proanthocyanidin, and other bitter metabolites in GG were not measured. The relationship between performance, carcass, meat quality parameters, and the profile of bitter metabolites in GG and caffeine should be evaluated in the future to better understand their effect on appetite, backfat deposition, and pork colour.

## 5. Conclusions

The dietary inclusion of GG in combination with caffeine reduced feed intake (short-term), whereas caffeine reduced ADG and backfat thickness in finishing pigs. The BM also increased the loin’s a* (redness), b* (yellowness), and C* (colour saturation) values. The use of higher doses of the BM led to a more pronounced (linear) reduction in ADFI, ADG, HSCW, dressing percentage, and backfat deposition in pigs and an increment in the pork yellowness, redness, and colour vividness. The changes in meat colour were associated primarily with an increased abundance of proteins involved in energy metabolism and myofibril assembly. Overall, the results show that GG could be used in combination with caffeine to modulate short-term appetite and improve carcass and pork quality, while also reducing the in-feed effective doses of caffeine.

## Figures and Tables

**Table 1:** Composition of experimental diets (as-fed basis).

	Con	Caf	BM 0.5 ^1^	BM 1.0	BM 1.5	BM 2.0	GG
Ingredients, %							
Wheat	46.72	46.72	46.72	46.72	46.72	46.72	46.72
Barley	35.00	35.00	35.00	35.00	35.00	35.00	35.00
Soybean Meal	2.50	2.50	2.50	2.50	2.50	2.50	2.50
Canola Meal	12.00	12.00	12.00	12.00	12.00	12.00	12.00
Water	1.00	1.00	1.00	1.00	1.00	1.00	1.00
Tallow mixer	0.33	0.33	0.33	0.33	0.33	0.33	0.33
Salt	0.50	0.50	0.50	0.50	0.50	0.50	0.50
Limestone	1.10	1.10	1.10	1.10	1.10	1.10	1.10
DL-Methionine	0.05	0.05	0.05	0.05	0.05	0.05	0.05
Caffeine	-	0.05	0.0125	0.025	0.0375	0.05	-
Grape seed extract	-	-	0.01875	0.0375	0.0562	0.075	0.075
Gentian plant extract	-	-	0.01875	0.0375	0.0562	0.075	0.075
Lysine-HCL	0.47	0.47	0.47	0.47	0.47	0.47	0.47
Threonine	0.15	0.15	0.15	0.15	0.15	0.15	0.15
Premix ^2^	0.15	0.15	0.15	0.15	0.15	0.15	0.15
Calculated nutrients, %							
Dry matter	89.19	89.19	89.19	89.19	89.19	89.19	89.19
Digestible energy, MJ/kg	13.83	13.83	13.83	13.83	13.83	13.83	13.83
Crude protein	14.64	14.64	14.64	14.64	14.64	14.64	14.64
Ether extract	2.28	2.28	2.28	2.28	2.28	2.28	2.28
Fibre	3.82	3.82	3.82	3.82	3.82	3.82	3.82
Ash	4.06	4.06	4.06	4.06	4.06	4.06	4.06
Available Phosphorus	0.40	0.40	0.40	0.40	0.40	0.40	0.40
Available Calcium	0.55	0.55	0.55	0.55	0.55	0.55	0.55
Available Lysine	0.86	0.86	0.86	0.86	0.86	0.86	0.86

Con, control diet; Caf, caffeine supplemented diet; BM, bitter mix (gentian, grape seed extracts and caffeine mixture) supplemented diet; GG, gentian plant and grape seed extract supplemented diet. ^1^ Concentration in the diet (g/kg). ^2^ Supplied per kilogram of diet: vitamin A, 8420 IU; vitamin D3, 1578 IU; vitamin E, 26.3 IU; vitamin K, 1.1 mg; vitamin B1, 1.1 mg; vitamin B2, 4.3 mg; vitamin B6, 1.6 mg; vitamin B12, 10.6 mg; niacin, 15.8 mg; pantothenic acid, 36.8 mg; biotin, 0.1 mg; iron, 63.2 mg; iodine, 0.5 mg; manganese, 63.2 mg; selenium, 0.3 mg; zinc, 126.3 mg; cobalt, 0.3 mg; chromium, 0.2 mg; copper, 21.9 mg.

**Table 2:** Performance and carcass quality of finishing pigs fed bitter compounds.

		Dietary Treatments					*p*-Value		
	Parameter	Control	Caffeine ^1^	BM ^2^	GG ^3^	SEM	Treat	Sex	Sex x Treat
Day 0	Initial weight (kg)	66.16	67.00	67.67	66.60	7.948	0.591	0.123	0.397
Day 42	Final weight (kg)	104.81	101.33	102.45	105.80	1.320	0.052	0.001	0.826
Day 0–21									
	ADFI (kg/d)	2.21 ^a^	2.13 ^ab^	1.97 ^b^	2.20 ^a^	0.047	0.003	0.337	0.471
	ADG (kg/d)	0.82	0.78	0.73	0.84	0.031	0.069	0.135	0.867
	FCR	2.68	2.72	2.77	2.53	0.064	0.082	0.002	0.371
Day 21–42									
	ADFI (kg/d)	2.53	2.39	2.47	2.55	0.102	0.618	0.007	0.721
	ADG (kg/d)	1.03	0.92	0.96	1.02	0.036	0.126	0.007	0.730
	FCR	2.60	2.47	2.50	2.55	0.116	0.868	0.583	0.792
Day 0–42									
	ADFI (kg/d)	2.45	2.30	2.27	2.41	0.063	0.145	0.073	0.557
	ADG (kg/d)	0.96 ^a^	0.88 ^b^	0.88 ^b^	0.96 ^a^	0.019	0.003	<0.001	0.365
	FCR	2.59	2.56	2.53	2.54	0.069	0.938	0.085	0.611
Carcass quality									
	HSCW (kg)	78.96	76.60	76.63	78.86	0.731	0.024	0.691	0.808
	P2 backfat (mm)	11.10 ^a^	10.38 ^b^	10.40 ^b^	11.05 ^ab^	0.173	0.002	0.356	0.158
	Loin depth (mm)	54.86	53.18	54.20	54.55	0.470	0.085	<0.001	0.155
	Dressing (%)	75.61	75.79	74.36	74.62	0.489	0.082	<0.001	0.499

BM, bitter mix; GG, gentian and grape seed extracts; Treat, dietary treatment effect; ADFI, average daily feed intake; ADG, average daily gain; FCR, feed conversion ratio; HSCW, hot standard carcass weight. ^1^ Supplemented at 0.5 g/kg. ^2^ Supplemented at 2 g/kg. The mixture contained caffeine (25%), gentian (37.5%), and grape seed extract (37.5%). ^3^ Supplemented at 1.5 g/kg. ^a,b^ Values within a row with different superscripts differ significantly at *p* < 0.05.

**Table 3:** Performance and carcass quality of finishing pigs fed increased dietary concentrations of a bitter mix.

		BM ^1^ (g/kg)						*p*-Value	
	Parameter	0 ^2^	0.5	1.0	1.5	2.0	SEM	Linear ^3^	Quadratic ^4^
Day 0	Initial weight (kg)	66.16	66.63	66.03	66.23	67.67	4.901	0.239	0.284
Day 42	Final weight (kg)	104.69	104.36	103.38	101.18	102.31	0.892	0.070	0.726
Day 0–21									
	ADFI (kg/day)	2.19	2.10	2.12	2.09	1.97	0.026	<0.001	0.459
	ADG (kg/day)	0.82	0.80	0.85	0.81	0.72	0.016	0.016	0.009
	FCR	2.68	2.65	2.51	2.70	2.70	0.039	0.722	0.057
Day 21–42									
	ADFI (kg/day)	2.59	2.59	2.48	2.37	2.39	0.064	0.063	0.838
	ADG (kg/day)	1.03	1.00	0.94	0.90	0.97	0.022	0.037	0.077
	FCR	2.57	2.69	2.64	2.66	2.48	0.086	0.669	0.258
Day 0–42									
	ADFI (kg/day)	2.43	2.44	2.39	2.26	2.26	0.044	0.031	0.827
	ADG (kg/day)	0.96	0.93	0.92	0.88	0.88	0.013	<0.001	0.564
	FCR	2.56	2.63	2.63	2.59	2.51	0.054	0.660	0.307
Carcass quality									
	HSCW (kg)	78.86	78.50	78.84	76.02	76.70	0.561	0.015	0.873
	P2 backfat (mm)	11.26	10.89	10.68	10.27	10.51	0.135	0.001	0.201
	Loin depth (mm)	54.79	54.27	53.67	52.90	54.06	0.397	0.269	0.110
	Dressing (%)	75.55	75.36	75.48	75.22	74.37	0.275	0.042	0.285

BM, bitter mix; ADFI, average daily feed intake; ADG, average daily gain; FCR, feed conversion ratio; HSCW, hot standard carcass weight. ^1^ Bitter mixture containing caffeine (25%), gentian (37.5%), and grape seed extract (37.5%). ^2^ Control diet with no bitter compounds. ^3^ Linear effect of increasing dietary levels of bitter mix. ^4^ Quadratic effect of increasing dietary levels of bitter mix. Statistically significant difference at *p* < 0.05.

**Table 4:** Quality attributes of the loin (longissimus thoracis muscle) in finishing pigs fed bitter compounds.

	Dietary Treatments					*p*-Value		
Parameter	Control	Caffeine	BM ^1^	GG ^2^	SEM	Treat ^3^	Sex	Sex x Treat
Drip loss (%)	3.59	4.26	4.60	3.17	0.006	0.284	0.448	0.206
pH 45 min	6.14	6.35	6.30	6.24	0.064	0.161	0.697	0.725
pH 24 h	5.51	5.46	5.50	5.50	0.027	0.466	0.655	0.323
Lightness (L*)	47.46	48.08	46.80	47.57	1.047	0.597	0.372	0.511
Redness (a*)	5.44	6.57	5.95	6.16	0.375	0.133	0.201	0.198
Yellowness (b*)	1.90	3.10	2.36	2.73	0.331	0.084	0.465	0.203
Hue angle ^3^ (h°)	19.59	25.03	21.55	22.35	2.545	0.421	0.797	0.406
Chroma ^4^ (C*)	5.78	7.27	6.41	6.80	0.432	0.082	0.193	0.174
Cook loss (%)	19.36	21.10	20.90	19.52	0.069	0.174	0.296	0.251
Shear force (N)	44.55	42.67	55.08	37.53	3.677	0.083	0.977	0.993

BM, bitter mix; GG, gentian and grape seed extracts; Treat, dietary treatment effect. ^1^ Supplemented at 2 g/kg. The mixture contained caffeine (25%), gentian (37.5%), and grape seed extract (37.5%). ^2^ Supplemented at 1.5 g/kg. ^3^ Hue angle = tan^−1^(b*/a*). ^4^ Chroma = (a*^2^ + b*^2^)^1/2^. Statistically significant difference at *p* < 0.05.

**Table 5:** Quality attributes of the loin (longissimus thoracis muscle) in finishing pigs fed increased dietary concentrations of a bitter mix.

	BM ^1^ (g/kg)						*p*-Value	
Parameter	0 ^2^	0.5	1.0	1.5	2.0	SEM	Linear ^3^	Quadratic ^4^
Drip loss (%)	3.60	3.10	3.55	5.11	4.60	0.005	0.328	0.694
pH 45 min	6.14	6.21	6.20	6.12	6.30	0.044	0.142	0.453
pH 24 h	5.52	5.56	5.48	5.41	5.50	0.023	0.351	0.267
Lightness (L*)	47.46	47.08	48.11	49.06	46.80	0.588	0.719	0.176
Redness (a*)	5.45	5.00	5.65	6.94	5.95	0.274	0.013	0.919
Yellowness (b*)	1.90	2.20	2.54	3.46	2.36	0.194	0.019	0.086
Hue angle ^5^ (h°)	19.59	24.00	24.18	26.47	21.55	1.194	0.315	0.020
Chroma ^6^ (C*)	5.78	5.50	6.20	7.77	6.41	0.309	0.010	0.740
Cook loss (%)	19.36	19.60	18.90	20.12	20.90	0.005	0.252	0.152
Shear force (N)	44.55	48.76	48.04	40.29	49.32	2.631	0.918	0.647

BM, bitter mix. ^1^ Bitter mixture containing caffeine (25%), gentian (37.5%), and grape seed extract (37.5%). ^2^ Control diet with no bitter compounds. ^3^ Linear effect of increasing dietary levels of bitter mix. ^4^ Quadratic effect of increasing dietary levels of bitter mix. ^5^ Hue angle = tan^−1^(b*/a*). ^6^ Chroma = (a*^2^ + b*^2^)^1/2^. Statistically significant difference at *p* < 0.05.

## Data Availability

All data generated during this study are available from the corresponding author upon reasonable request.

## Supplementary Materials

The following supporting information can be downloaded at: https://www.mdpi.com/article/10.3390/ani15142129/s1, Table S1: Gene ontology term enrichment of pork loin following the dietary supplementation of bitter compounds in finishing pigs.

## Abbreviations

The following abbreviations are used in the manuscript:

*a**	Redness
ADFI	Average daily feed intake
ADG	Average daily gain
ANOVA	Analysis of Variance
ATP	Adenosine triphosphate
*b**	Yellowness
BM	Bitter mix
DAP	Differentially abundant proteins
DIA-NN	data-independent acquisition neural network
FCR	Feed conversion ration
GG	Gentian and grape seed extract
GO	Gene Ontology
HSCW	Hot standard carcass weight
*L**	Lightness
SWATH	Sequential Windowed Acquisition of all Theoretical fragment ions
ZenoTOF	Zeno time-of-flight
