# Supplementary material for: Feeding a Bitter Mix of Gentian and Grape Seed Extracts with Caffeine Reduces Appetite and Body Fat Deposition and Improves Meat Colour in Pigs
Source: Animals (Basel). 2025 Jul 18;15(14):2129. doi: 10.3390/ani15142129 (PMC12291743; doi:10.3390/ani15142129)
Supplement: Supplementary file 1 [file animals-15-02129-s001.zip › animals-3746746-supplementary.pdf]

Supplementary Material

Table S1. Gene ontology term enrichment. Significantly (FDR < 0.05) up-regulated ontologies in *longissimus lumborum* muscle of bitter mix (combination of caffeine, grape and gentian extracts) fed pigs.

Gene Ontology ID	Term	Proteins	FDR
**Biological Process**			
GO:0055114	oxidation-reduction process	A0A024BTL2_PIG; A0A286ZIJ9_PIG; A0A480QR88_PIG; A0A4X1TPC0_PIG; A0A4X1UYT9_PIG; ACADS_PIG; COX41_PIG; COX5B_PIG; MDHM_PIG	0.0010
GO:0046034	ATP metabolic process	A0A076EBU5_PIG; A0A286ZIJ9_PIG; A0A480QR88_PIG; A0A4X1TPC0_PIG; A0A4X1UYT9_PIG; ATPA_PIG; COX41_PIG; COX5B_PIG	0.0010
GO:0009199	ribonucleoside triphosphate metabolic process	A0A076EBU5_PIG; A0A286ZIJ9_PIG; A0A480QR88_PIG; A0A4X1TPC0_PIG; A0A4X1UYT9_PIG; ATPA_PIG; COX41_PIG; COX5B_PIG	0.0010
GO:0009141	nucleoside triphosphate metabolic process	A0A076EBU5_PIG; A0A286ZIJ9_PIG; A0A480QR88_PIG; A0A4X1TPC0_PIG; A0A4X1UYT9_PIG; ATPA_PIG; COX41_PIG; COX5B_PIG	0.0010
GO:0009144	purine nucleoside triphosphate metabolic process	A0A076EBU5_PIG; A0A286ZIJ9_PIG; A0A480QR88_PIG; A0A4X1TPC0_PIG; A0A4X1UYT9_PIG; ATPA_PIG; COX41_PIG; COX5B_PIG	0.0010
GO:0009205	purine ribonucleoside triphosphate metabolic process	A0A076EBU5_PIG; A0A286ZIJ9_PIG; A0A480QR88_PIG; A0A4X1TPC0_PIG; A0A4X1UYT9_PIG; ATPA_PIG; COX41_PIG; COX5B_PIG	0.0010
GO:0009126	purine nucleoside monophosphate metabolic process	A0A076EBU5_PIG; A0A286ZIJ9_PIG; A0A480QR88_PIG; A0A4X1TPC0_PIG; A0A4X1UYT9_PIG; ATPA_PIG; COX41_PIG; COX5B_PIG	0.0013
GO:0009123	nucleoside monophosphate metabolic process	A0A076EBU5_PIG; A0A286ZIJ9_PIG; A0A480QR88_PIG; A0A4X1TPC0_PIG; A0A4X1UYT9_PIG; ATPA_PIG; COX41_PIG; COX5B_PIG	0.0013
GO:0009161	ribonucleoside monophosphate metabolic process	A0A076EBU5_PIG; A0A286ZIJ9_PIG; A0A480QR88_PIG; A0A4X1TPC0_PIG; A0A4X1UYT9_PIG; ATPA_PIG; COX41_PIG; COX5B_PIG	0.0013
GO:0009167	purine ribonucleoside monophosphate metabolic process	A0A076EBU5_PIG; A0A286ZIJ9_PIG; A0A480QR88_PIG; A0A4X1TPC0_PIG; A0A4X1UYT9_PIG; ATPA_PIG; COX41_PIG; COX5B_PIG	0.0013
GO:0009150	purine ribonucleotide metabolic process	A0A076EBU5_PIG; A0A286ZIJ9_PIG; A0A480QR88_PIG; A0A4X1TPC0_PIG; A0A4X1UYT9_PIG; ATPA_PIG; COX41_PIG; COX5B_PIG	0.0037
GO:0009259	ribonucleotide metabolic process	A0A076EBU5_PIG; A0A286ZIJ9_PIG; A0A480QR88_PIG; A0A4X1TPC0_PIG; A0A4X1UYT9_PIG; ATPA_PIG; COX41_PIG; COX5B_PIG	0.0037
GO:0019693	ribose phosphate metabolic process	A0A076EBU5_PIG; A0A286ZIJ9_PIG; A0A480QR88_PIG; A0A4X1TPC0_PIG; A0A4X1UYT9_PIG; ATPA_PIG; COX41_PIG; COX5B_PIG	0.0038
GO:0006163	purine nucleotide metabolic process	A0A076EBU5_PIG; A0A286ZIJ9_PIG; A0A480QR88_PIG; A0A4X1TPC0_PIG; A0A4X1UYT9_PIG; ATPA_PIG; COX41_PIG; COX5B_PIG	0.0038
GO:0072521	purine-containing compound metabolic process	A0A076EBU5_PIG; A0A286ZIJ9_PIG; A0A480QR88_PIG; A0A4X1TPC0_PIG; A0A4X1UYT9_PIG; ATPA_PIG; COX41_PIG; COX5B_PIG	0.0048
GO:0015980	energy derivation by oxidation of organic compounds	A0A024BTL2_PIG; A0A480QR88_PIG; A0A4X1TPC0_PIG; A0A4X1UYT9_PIG; COX41_PIG; COX5B_PIG; MDHM_PIG	0.0048
GO:0042775	mitochondrial ATP synthesis coupled electron transport	A0A480QR88_PIG; A0A4X1TPC0_PIG; A0A4X1UYT9_PIG; COX41_PIG; COX5B_PIG	0.0048

GO:0006753	nucleoside phosphate metabolic process	A0A076EBU5_PIG; A0A286ZIJ9_PIG; A0A480QR88_PIG; A0A4X1TPC0_PIG; A0A4X1UYT9_PIG; ATPA_PIG; COX41_PIG; COX5B_PIG	0.0048
GO:0009117	nucleotide metabolic process	A0A076EBU5_PIG; A0A286ZIJ9_PIG; A0A480QR88_PIG; A0A4X1TPC0_PIG; A0A4X1UYT9_PIG; ATPA_PIG; COX41_PIG; COX5B_PIG	0.0048
GO:0019637	organophosphate metabolic process	A0A076EBU5_PIG; A0A286ZIJ9_PIG; A0A480QR88_PIG; A0A4X1T6K3_PIG; A0A4X1TPC0_PIG; A0A4X1UYT9_PIG; ATPA_PIG; COX41_PIG; COX5B_PIG	0.0054
GO:0042773	ATP synthesis coupled electron transport	A0A480QR88_PIG; A0A4X1TPC0_PIG; A0A4X1UYT9_PIG; COX41_PIG; COX5B_PIG	0.0061
GO:0006091	generation of precursor metabolites and energy	A0A024BTL2_PIG; A0A286ZIJ9_PIG; A0A480QR88_PIG; A0A4X1TPC0_PIG; A0A4X1UYT9_PIG; COX41_PIG; COX5B_PIG; MDHM_PIG	0.0067
GO:0045333	cellular respiration	A0A480QR88_PIG; A0A4X1TPC0_PIG; A0A4X1UYT9_PIG; COX41_PIG; COX5B_PIG; MDHM_PIG	0.0072
GO:0055086	nucleobase-containing small molecule metabolic process	A0A076EBU5_PIG; A0A286ZIJ9_PIG; A0A480QR88_PIG; A0A4X1TPC0_PIG; A0A4X1UYT9_PIG; ATPA_PIG; COX41_PIG; COX5B_PIG	0.0072
GO:0017144	drug metabolic process	A0A076EBU5_PIG; A0A286ZIJ9_PIG; A0A480QR88_PIG; A0A4X1TPC0_PIG; A0A4X1UYT9_PIG; ATPA_PIG; COX41_PIG; COX5B_PIG; MDHM_PIG	0.0074
GO:0022900	electron transport chain	A0A480QR88_PIG; A0A4X1TPC0_PIG; A0A4X1UYT9_PIG; COX41_PIG; COX5B_PIG	0.0088
GO:0022904	respiratory electron transport chain	A0A480QR88_PIG; A0A4X1TPC0_PIG; A0A4X1UYT9_PIG; COX41_PIG; COX5B_PIG	0.0088
GO:0006119	oxidative phosphorylation	A0A480QR88_PIG; A0A4X1TPC0_PIG; A0A4X1UYT9_PIG; COX41_PIG; COX5B_PIG	0.0107
GO:0019646	aerobic electron transport chain	A0A4X1TPC0_PIG; COX41_PIG; COX5B_PIG	0.0107
GO:0006123	mitochondrial electron transport, cytochrome c to oxygen	A0A4X1TPC0_PIG; COX41_PIG; COX5B_PIG	0.0107
GO:1901135	carbohydrate derivative metabolic process	A0A076EBU5_PIG; A0A286ZIJ9_PIG; A0A480QR88_PIG; A0A4X1TPC0_PIG; A0A4X1UYT9_PIG; ATPA_PIG; COX41_PIG; COX5B_PIG	0.0114
GO:0003012	muscle system process	MYH2_PIG; MYH4_PIG; MYH7_PIG; MYOZ1_PIG; TNNT1_PIG	0.0163
GO:0045214	sarcomere organization	MYH7_PIG; MYOZ1_PIG; TNNT1_PIG	0.0185
GO:1902600	proton transmembrane transport	A0A076EBU5_PIG; AT1A2_PIG; ATPA_PIG	0.0185
GO:0048468	cell development	A0A287BCF1_PIG; A0A4X1UYT9_PIG; MYH7_PIG; MYOZ1_PIG; TNNT1_PIG	0.0234
GO:0009060	aerobic respiration	A0A4X1TPC0_PIG; COX41_PIG; COX5B_PIG; MDHM_PIG	0.0252
GO:0006936	muscle contraction	MYH2_PIG; MYH4_PIG; MYH7_PIG;TNNT1_PIG	0.0422
GO:0015672	monovalent inorganic cation transport	A0A076EBU5_PIG; AT1A2_PIG; ATPA_PIG	0.0439
**Cellular Component**			
GO:0016459	myosin complex	A0A286ZPQ9_PIG; A0A8W4FFN5_PIG; MYH2_PIG; MYH4_PIG; MYH7_PIG	0.0032
GO:0098800	inner mitochondrial membrane protein complex	A0A076EBU5_PIG; A0A480QR88_PIG; A0A4X1TPC0_PIG; A0A4X1UYT9_PIG; ATPA_PIG; COX41_PIG; COX5B_PIG	0.0037
GO:0070069	cytochrome complex	A0A4X1TPC0_PIG; A0A4X1UYT9_PIG; COX41_PIG; COX5B_PIG	0.0037
GO:0044455	mitochondrial membrane part	A0A076EBU5_PIG; A0A480QR88_PIG; A0A4X1TPC0_PIG; A0A4X1UYT9_PIG; ATPA_PIG; COX41_PIG; COX5B_PIG	0.0037
GO:0098798	mitochondrial protein complex	A0A076EBU5_PIG; A0A480QR88_PIG; A0A4X1TPC0_PIG; A0A4X1UYT9_PIG; ATPA_PIG; COX41_PIG; COX5B_PIG	0.0049
GO:0015629	actin cytoskeleton	A0A286ZPQ9_PIG; A0A8W4FFN5_PIG; MYH2_PIG; MYH4_PIG; MYH7_PIG; MYOZ1_PIG; TNNT1_PIG	0.0049
GO:0030016	myofibril	A0A286ZPQ9_PIG; MYH2_PIG; MYH4_PIG; MYH7_PIG; MYOZ1_PIG; TNNT1_PIG	0.0078

GO:0043292	contractile fiber	A0A286ZPQ9_PIG; MYH2_PIG; MYH4_PIG; MYH7_PIG; MYOZ1_PIG; TNNT1_PIG	0.0078
GO:0032982	myosin filament	MYH2_PIG; MYH4_PIG; MYH7_PIG	0.0078
GO:0045277	respiratory chain complex IV	A0A4X1TPC0_PIG; COX41_PIG; COX5B_PIG	0.0078
GO:0005751	mitochondrial respiratory chain complex IV	A0A4X1TPC0_PIG; COX41_PIG; COX5B_PIG	0.0078
GO:0016460	myosin II complex	MYH2_PIG;MYH4_PIG;MYH7_PIG	0.0078
GO:0005746	mitochondrial respiratory chain	A0A480QR88_PIG; A0A4X1TPC0_PIG; A0A4X1UYT9_PIG; COX41_PIG; COX5B_PIG	0.0133
GO:0098796	membrane protein complex	A0A076EBU5_PIG; A0A480QR88_PIG; A0A4X1TPC0_PIG; A0A4X1UYT9_PIG; AT1A2_PIG; ATPA_PIG; COX41_PIG; COX5B_PIG	0.0133
GO:0098803	respiratory chain complex	A0A480QR88_PIG; A0A4X1TPC0_PIG; A0A4X1UYT9_PIG; COX41_PIG; COX5B_PIG	0.0153
GO:0044429	mitochondrial part	A0A076EBU5_PIG; A0A480QR88_PIG; A0A4X1TPC0_PIG; A0A4X1UYT9_PIG; ACADS_PIG; ATPA_PIG; COX41_PIG; COX5B_PIG; MDHM_PIG	0.0207
GO:0019866	organelle inner membrane	A0A076EBU5_PIG; A0A480QR88_PIG; A0A4X1TPC0_PIG; A0A4X1UYT9_PIG; ATPA_PIG; COX41_PIG; COX5B_PIG	0.0219
GO:0005743	mitochondrial inner membrane	A0A076EBU5_PIG; A0A480QR88_PIG; A0A4X1TPC0_PIG; A0A4X1UYT9_PIG; ATPA_PIG; COX41_PIG; COX5B_PIG	0.0219
GO:0070469	respiratory chain	A0A480QR88_PIG; A0A4X1TPC0_PIG; A0A4X1UYT9_PIG; COX41_PIG; COX5B_PIG	0.0341
GO:0061695	transferase complex, transferring phosphorus-containing groups	A0A024BTL2_PIG; A0A286ZIJ9_PIG	0.0341
GO:0032991	protein-containing complex	A0A024BTL2_PIG; A0A076EBU5_PIG; A0A286ZIJ9_PIG; A0A286ZPQ9_PIG; A0A480QR88_PIG; A0A4X1TPC0_PIG; A0A4X1UYT9_PIG; A0A4X1VT96_PIG; A0A8W4FFN5_PIG; AT1A2_PIG; ATPA_PIG; COX41_PIG; COX5B_PIG; MYH2_PIG; MYH4_PIG; MYH7_PIG; TNNT1_PIG	0.0341
GO:0031966	mitochondrial membrane	A0A076EBU5_PIG; A0A480QR88_PIG; A0A4X1TPC0_PIG; A0A4X1UYT9_PIG; ATPA_PIG; COX41_PIG; COX5B_PIG	0.0491
**Molecular Function**			
GO:0003774	motor activity	A0A286ZPQ9_PIG; A0A287BCF1_PIG; A0A8W4FFN5_PIG; MYH2_PIG; MYH4_PIG; MYH7_PIG	0.0002
GO:0000146	microfilament motor activity	MYH2_PIG; MYH4_PIG; MYH7_PIG	0.0064
GO:0008092	cytoskeletal protein binding	A0A286ZPQ9_PIG; A0A287BCF1_PIG; A0A481BBQ7_PIG; A0A8W4FFN5_PIG; MYH2_PIG; MYH4_PIG; MYH7_PIG; MYOZ1_PIG; TNNT1_PIG	0.0064
GO:0005516	calmodulin binding	A0A024BTL2_PIG; MYH2_PIG; MYH4_PIG; MYH7_PIG	0.0064
GO:0051015	actin filament binding	A0A286ZPQ9_PIG; A0A8W4FFN5_PIG; MYH2_PIG; MYH4_PIG; MYH7_PIG	0.0067
GO:0003779	actin binding	A0A286ZPQ9_PIG; A0A481BBQ7_PIG; A0A8W4FFN5_PIG; MYH2_PIG; MYH4_PIG; MYH7_PIG; MYOZ1_PIG	0.0090
GO:0005524	ATP binding	A0A024BTL2_PIG; A0A286ZIJ9_PIG; A0A286ZPQ9_PIG; A0A287BCF1_PIG; A0A4X1T6K3_PIG; A0A8W4FFN5_PIG; AT1A2_PIG; ATPA_PIG; MYH2_PIG; MYH4_PIG; MYH7_PIG	0.0106
GO:0032559	adenyl ribonucleotide binding	A0A024BTL2_PIG; A0A286ZIJ9_PIG; A0A286ZPQ9_PIG; A0A287BCF1_PIG; A0A4X1T6K3_PIG; A0A8W4FFN5_PIG; AT1A2_PIG; ATPA_PIG; MYH2_PIG; MYH4_PIG; MYH7_PIG	0.0106
GO:0030554	adenyl nucleotide binding	A0A024BTL2_PIG; A0A286ZIJ9_PIG; A0A286ZPQ9_PIG; A0A287BCF1_PIG; A0A4X1T6K3_PIG; A0A8W4FFN5_PIG; AT1A2_PIG; ATPA_PIG; MYH2_PIG; MYH4_PIG; MYH7_PIG	0.0106
GO:0008144	drug binding	A0A024BTL2_PIG; A0A286ZIJ9_PIG; A0A286ZPQ9_PIG; A0A287BCF1_PIG; A0A4X1T6K3_PIG; A0A8W4FFN5_PIG; AT1A2_PIG; ATPA_PIG; MYH2_PIG; MYH4_PIG; MYH7_PIG	0.0129
GO:0035639	purine ribonucleoside triphosphate binding	A0A024BTL2_PIG; A0A286ZIJ9_PIG; A0A286ZPQ9_PIG; A0A287BCF1_PIG; A0A4X1T6K3_PIG; A0A4X1VRP4_PIG;	0.0147

		A0A8W4FFN5_PIG; AT1A2_PIG; ATPA_PIG; MYH2_PIG; MYH4_PIG; MYH7_PIG	
GO:0032555	purine ribonucleotide binding	A0A024BTL2_PIG; A0A286ZIJ9_PIG; A0A286ZPQ9_PIG; A0A287BCF1_PIG; A0A4X1T6K3_PIG; A0A4X1VRP4_PIG; A0A8W4FFN5_PIG; AT1A2_PIG; ATPA_PIG; MYH2_PIG; MYH4_PIG; MYH7_PIG	0.0150
GO:0017076	purine nucleotide binding	A0A024BTL2_PIG; A0A286ZIJ9_PIG; A0A286ZPQ9_PIG; A0A287BCF1_PIG; A0A4X1T6K3_PIG; A0A4X1VRP4_PIG; A0A8W4FFN5_PIG; AT1A2_PIG; ATPA_PIG; MYH2_PIG; MYH4_PIG; MYH7_PIG	0.0150
GO:0032553	ribonucleotide binding	A0A024BTL2_PIG; A0A286ZIJ9_PIG; A0A286ZPQ9_PIG; A0A287BCF1_PIG; A0A4X1T6K3_PIG; A0A4X1VRP4_PIG; A0A8W4FFN5_PIG; AT1A2_PIG; ATPA_PIG; MYH2_PIG; MYH4_PIG; MYH7_PIG	0.0150
GO:0005515	protein binding	A0A024BTL2_PIG; A0A286ZIJ9_PIG; A0A286ZPQ9_PIG; A0A287BCF1_PIG; A0A481BBQ7_PIG; A0A8W4FFN5_PIG; HPT_PIG; MDHM_PIG; MYH2_PIG; MYH4_PIG; MYH7_PIG; MYOZ1_PIG; TNNT1_PIG	0.0162
GO:1901265	nucleoside phosphate binding	A0A024BTL2_PIG; A0A286ZIJ9_PIG; A0A286ZPQ9_PIG; A0A287BCF1_PIG; A0A4X1T6K3_PIG; A0A4X1VRP4_PIG; A0A8W4FFN5_PIG; ACADS_PIG; AT1A2_PIG; ATPA_PIG; MYH2_PIG; MYH4_PIG; MYH7_PIG	0.0179
GO:0000166	nucleotide binding	A0A024BTL2_PIG; A0A286ZIJ9_PIG; A0A286ZPQ9_PIG; A0A287BCF1_PIG; A0A4X1T6K3_PIG; A0A4X1VRP4_PIG; A0A8W4FFN5_PIG; ACADS_PIG; AT1A2_PIG; ATPA_PIG; MYH2_PIG; MYH4_PIG; MYH7_PIG	0.0179
GO:0097367	carbohydrate derivative binding	A0A024BTL2_PIG; A0A286ZIJ9_PIG; A0A286ZPQ9_PIG; A0A287BCF1_PIG; A0A4X1T6K3_PIG; A0A4X1VRP4_PIG; A0A8W4FFN5_PIG; AT1A2_PIG; ATPA_PIG; MYH2_PIG; MYH4_PIG; MYH7_PIG	0.0189
GO:0043168	anion binding	A0A024BTL2_PIG; A0A286ZIJ9_PIG; A0A286ZPQ9_PIG; A0A287BCF1_PIG; A0A4X1T6K3_PIG; A0A4X1VRP4_PIG; A0A8W4FFN5_PIG; ACADS_PIG; AT1A2_PIG; ATPA_PIG; MYH2_PIG; MYH4_PIG; MYH7_PIG	0.0261
GO:0036094	small molecule binding	A0A024BTL2_PIG; A0A286ZIJ9_PIG; A0A286ZPQ9_PIG; A0A287BCF1_PIG; A0A4X1T6K3_PIG; A0A4X1VRP4_PIG; A0A8W4FFN5_PIG; ACADS_PIG; AT1A2_PIG; ATPA_PIG; MYH2_PIG; MYH4_PIG; MYH7_PIG	0.0272
GO:0017111	nucleoside-triphosphatase activity	A0A286ZPQ9_PIG; A0A287BCF1_PIG; A0A8W4FFN5_PIG; AT1A2_PIG; ATPA_PIG; MYH2_PIG; MYH4_PIG; MYH7_PIG	0.0272
GO:0016462	pyrophosphatase activity	A0A286ZPQ9_PIG; A0A287BCF1_PIG; A0A8W4FFN5_PIG; AT1A2_PIG; ATPA_PIG; MYH2_PIG; MYH4_PIG; MYH7_PIG	0.0296
GO:0016817	hydrolase activity, acting on acid anhydrides	A0A286ZPQ9_PIG; A0A287BCF1_PIG; A0A8W4FFN5_PIG; AT1A2_PIG; ATPA_PIG; MYH2_PIG; MYH4_PIG; MYH7_PIG	0.0296
GO:0016818	hydrolase activity, acting on acid anhydrides, in phosphorus-containing anhydrides	A0A286ZPQ9_PIG; A0A287BCF1_PIG; A0A8W4FFN5_PIG; AT1A2_PIG; ATPA_PIG; MYH2_PIG; MYH4_PIG; MYH7_PIG	0.0296
GO:0044877	protein-containing complex binding	A0A286ZPQ9_PIG; A0A8W4FFN5_PIG; MYH2_PIG; MYH4_PIG; MYH7_PIG	0.0296
GO:0015077	monovalent inorganic cation transmembrane transporter activity	A0A076EBU5_PIG; AT1A2_PIG; ATPA_PIG	0.0325
